# Evidence for the Convergence Model: The Emergence of Highly Pathogenic Avian Influenza (H5N1) in Viet Nam

**DOI:** 10.1371/journal.pone.0138138

**Published:** 2015-09-23

**Authors:** Sumeet Saksena, Jefferson Fox, Michael Epprecht, Chinh C. Tran, Duong H. Nong, James H. Spencer, Lam Nguyen, Melissa L. Finucane, Vien D. Tran, Bruce A. Wilcox

**Affiliations:** 1 East-West Center, Honolulu, Hawaii, United States of America; 2 University of Bern, Bern, Switzerland; 3 Clemson University, Clemson, South Carolina, United States of America; 4 Vietnam National University of Agriculture, Hanoi, Vietnam; 5 Rand Corporation, Pittsburgh, Pennsylvania, United States of America; 6 Mahihdol University, Bangkok, Thailand; Rutgers University, UNITED STATES

## Abstract

Building on a series of ground breaking reviews that first defined and drew attention to emerging infectious diseases (EID), the ‘convergence model’ was proposed to explain the multifactorial causality of disease emergence. The model broadly hypothesizes disease emergence is driven by the co-incidence of genetic, physical environmental, ecological, and social factors. We developed and tested a model of the emergence of highly pathogenic avian influenza (HPAI) H5N1 based on suspected convergence factors that are mainly associated with land-use change. Building on previous geospatial statistical studies that identified natural and human risk factors associated with urbanization, we added new factors to test whether causal mechanisms and pathogenic landscapes could be more specifically identified. Our findings suggest that urbanization spatially combines risk factors to produce particular types of peri-urban landscapes with significantly higher HPAI H5N1 emergence risk. The work highlights that peri-urban areas of Viet Nam have higher levels of chicken densities, duck and geese flock size diversities, and fraction of land under rice or aquaculture than rural and urban areas. We also found that land-use diversity, a surrogate measure for potential mixing of host populations and other factors that likely influence viral transmission, significantly improves the model’s predictability. Similarly, landscapes where intensive and extensive forms of poultry production overlap were found at greater risk. These results support the convergence hypothesis in general and demonstrate the potential to improve EID prevention and control by combing geospatial monitoring of these factors along with pathogen surveillance programs.

## Introduction

Two decades after the Institute of Medicine’s seminal report [1] recognized novel and re-emerging diseases as a new category of microbial threats, the perpetual and unexpected nature of the emergence of infectious diseases remains a challenge in spite of significant clinical and biomedical research advances [2]. Highly Pathogenic Avian Influenza (HPAI) (subtype H5N1) is the most significant newly emerging pandemic disease since HIV/AIDS. Its eruption in Southeast Asia in 2003–4 and subsequent spread globally to more than 60 countries fits the complex systems definition of “surprise” [3]. In this same year that IOM had published its final report on microbial threats which highlighted H5N1's successful containment in Hong Kong in 1997 [4], massive outbreaks occurred in Southeast Asia where it remains endemic, along with Egypt’s Nile Delta. Since 2003, HPAI H5N1 has killed millions of poultry in countries throughout Asia, Europe, and Africa, and 402 humans have died from it in sixteen countries according to WHO data as of January 2015. The threat of a pandemic resulting in millions of human cases worldwide remains a possibility [5].

Lederberg et al. [1] first pointed to the multiplicity of factors driving disease emergence, which later were elaborated and described in terms of ‘the convergence model’ [6]. The model proposes emergence events are precipitated by the intensifying of biological, environmental, ecological, and socioeconomic drivers. Microbial “adaptation and change,” along with “changing ecosystems” and “economic development and land use” form major themes. Joshua Lederberg, the major intellectual force behind the studies summed-up saying “Ecological instabilities arise from the ways we alter the physical and biological environment, the microbial and animal tenants (humans included) of these environments, and our interactions (including hygienic and therapeutic interventions) with the parasites” [6].

Combining such disparate factors and associated concepts from biomedicine, ecology, and social sciences in a single framework remains elusive. One approach suggested has been to employ social-ecological systems theory that attempts to capture the behavior of so-called ‘coupled natural-human systems’, including the inevitable unexpected appearance of new diseases, themselves one of the “emerging properties” of complex adaptive systems (CAS) [7, 8]. The convergence model can be so adapted by incorporating the dynamics of urban, agricultural, and natural ecosystem transformations proposed with this framework. These associated multifaceted interactions including feedbacks that affect ecological communities, hosts and pathogen populations, are the proximate drivers of disease emergence.

The initial HPAI H5N1 outbreaks in Vietnam represent an ideal opportunity to adapt and test a CAS-convergence model. Emergence risk should be highest in the most rapidly transforming urban areas, peri-urban zones where mixes of urban-rural, modern-traditional land uses and poultry husbandry coincide most intensely. Specifically we hypothesized a positive association between the presence of HPAI outbreaks in poultry at the commune level and: 1) peri-urban areas, as defined by Saksena et al. [9], 2) land-use diversity, and 3) co-location of intensive and extensive systems of poultry.

We used the presence or absence at the commune level of HPAI H5N1 outbreaks in poultry as the dependent variable. Vietnam experienced its first HPAI H5N1 outbreak in late 2003, since then, there have been five waves and sporadic outbreaks recorded over the years [10, 11]. We chose to study the first wave (Wave 1) that ended in February 2004 and the second wave (Wave 2) that occurred between December 2004 and April 2005. We used data from the Viet Nam 2006 Agricultural Census to develop an urbanicity classification that used data collected at a single point in time (2006) but across space (10,820 communes) to infer processes of change (urbanization, land-use diversification, and poultry intensification) [9]. The 58 provinces in Vietnam (not counting the 5 urban provinces that are governed centrally) are divided into rural districts, provincial towns, and provincial cities. Rural districts are further divided into communes (rural areas) and towns, and provincial towns and cities are divided into wards (urban subdistricts) and communes. A commune in Viet Nam is thus the third level administrative subdivision, consisting of villages/hamlets. For the purpose of simplicity we will henceforth use the term “commune” to refer to the smallest administrative unit whether it is a commune, town, or ward. We included risk factors documented in previous work. We also aimed to understand the differences, if any, in risk dynamics at different scales; comparing risks at the national scale to those at two sub-national agro-ecological zones. For this purpose we chose to study the Red River and Mekong River deltas, well known hot spots of the disease. Hence we conducted two sets of analyses (waves 1 and 2) for three places (nation, Red River Delta, and Mekong Delta) producing a total of 6 wave-place analyses. Data on outbreaks were obtained from the publicly available database of Viet Nam’s Department of Animal Health. Given the highly complex dynamics of the epidemics and in keeping with recent methodological trends, we used multiple modeling approaches—parametric and non-parametric—with a focus on spatial analysis. We used both ‘place’ oriented models that can take into account variations in factors such as policies and administration as well as ‘space’ oriented models that recognize the importance of physical proximity in natural phenomenon [12].

### Urbanization

Very few empirical studies have attempted to determine whether urbanization is related to EID outbreaks or whether urbanization is associated primarily with other factors related to EID outbreaks. One immediate problem researchers face is defining what is rural, urban, and transitional (i.e., peri-urban). Some studies have used official administrative definitions of urban and rural areas, but this approach is limited in its bluntness [13]. Other studies prioritized human population density as a satisfactory surrogate [11, 14–20], but this approach ignores the important fact that density is not a risk factor if it is accompanied by sufficient infrastructure to handle the population. Spencer [21] examined urbanization as a non-linear characteristic, using household-level variables such as water and sanitation services. He found evidence that increased diversity in water supply sources and sanitation infrastructure were associated with higher incidences of HPAI. These studies employed a limited definition of urbanization that lacked a well-defined characterization of peri-urbanization.

Still other studies have mapped the relative urban nature of a place, a broad concept that is often referred to as ‘urbanicity' [22–25]. While these studies show differences in the rural/urban nature of communities across space and time, they have been limited to small- to medium-scale observational studies; and they have failed to distinguish between different levels of “ruralness”. Perhaps the best known model of peri-urbanization is McGee’s concept of desakota (Indonesian for “village-town”) [26]. McGee identified six characteristics of desakota regions: 1) a large population of smallholder cultivators; 2) an increase in non-agricultural activities; 3) extreme fluidity and mobility of population; 4) a mixture of land uses, agriculture, cottage industries, suburban development; 5) increased participation of the female labor force; and 6) “grey-zones”, where informal and illegal activities group [26]. Saksena et al. [9] built on McGee’s desakota concepts and data from the 2006 Viet Nam Agricultural Census to establish an urbanicity classification. That study identified and mapped the 10,820 communes, the smallest administrative unit for which data are collected, as being rural, peri-urban, urban, or urban core. This project used the Saksena classification to assess associations between urbanicity classes, other risks factors, and HPAI outbreaks.

### Land-use diversification

Researchers have estimated that almost 75% of zoonotic diseases are associated with land-cover and land-use changes (LCLUC) [27, 28]. LCLUC such as peri-urbanization and agricultural diversification frequently result in more diverse and fragmented landscapes (number of land covers or land uses per unit of land). The importance of landscape pattern, including diversity and associated processes, which equate to host species’ habitat size and distribution, and thus pathogen transmission dynamics is axiomatic though the specific mechanisms depend on the disease [29, 30]. Landscape fragmentation produces ecotones, defined as abrupt edges or transitions zones between different ecological systems, thought to facilitate disease emergence by increasing the intensity and frequency of contact between host species [31] Furthermore, fragmentation of natural habitat tends to interrupt and degrade natural processes, including interspecies interactions that regulate densities of otherwise opportunistic species that may serve as competent hosts [32], although it is not clear if reduced species diversity necessarily increases pathogen transmission [33]. Rarely has research connected land-use diversification to final health endpoints in humans or livestock; this study attempts to link land-use diversity with HPAI H5N1 outbreaks.

### Poultry Intensification

Human populations in the rapidly urbanizing cities of the developing world require access to vegetables, fruits, meat, etc. typically produced elsewhere. As theorized by von Thünen in 1826 [34], much of this demand is met by farms near cities [35], many in areas undergoing processes of peri-urbanization [26]. Due to the globalization of poultry trade, large-scale chicken farms raising thousands of birds have expanded rapidly in Southeast Asia and compete with existing small backyard farmers [36]. Large, enterprise-scale (15,000–100,000 birds) operations are still rare in Viet Nam (only 33 communes have such a facility). On the other hand, domestic and multinational companies frequently contract farmers to raise between 2,000 and 15,000 birds.

Recent studies have examined the relative role of extensive (backyard) systems and intensive systems [15, 17–19, 37]. In much of Asia there is often a mix of commercial and backyard farming at any one location [36]. Experts have suggested that from a biosecurity perspective the co-location of extensive and intensive systems is a potential risk factor [38]. Intensive systems allow for virus evolution (e.g. Low Pathogenic Avian Influenza to HPAI) and transformation, while extensive systems allow for environmental persistence and circulation [39]. Previous studies of chicken populations as a risk factor have distinguished between production systems—native chickens, backyard chickens; flock density; commercial chickens, broilers and layers density, etc. [15, 17–19, 37]. In isolation, however, none of these number and/or density based poultry metrics adequately measures the extent of co-location of intensive and extensive systems in any given place. Intensive and extensive systems in Viet Nam have their own fairly well defined flock sizes. A diversity index of the relative number of intensive and extensive systems of poultry- raising can better estimate the effect of such co-location; this study attempts to link a livestock diversity index with the presence or absence of HPAI H5N1 outbreaks at the commune level.

## Methods

This study investigated for the 10,820 communes of Viet Nam a wide suite of socio-economic, agricultural, climatic and ecological variables relevant to poultry management and the transmission and persistence of the HPAI virus. Many of these variables were identified based on earlier studies of HPAI (as reviewed in Gilbert and Pfeiffer [40]). Three novel variables were included based on hypotheses generated by this project. All variables were measured or aggregated to the commune level. The novel variables were:

- Degree of urbanization: We used the urbanicity classification developed by Saksena et al. [9] to define the urban character of each commune. The classification framework is based on four characteristics: 1) percentage of households whose main income is from agriculture, aquaculture and forestry, 2) percentage of households with modern forms of toilets, 3) percentage of land under agriculture, aquaculture and forestry and 4) the Normalized Differentiated Vegetation Index (NDVI). The three-way classification enabled testing for non-linear and non-monotonous responses.
- Land-use diversity: We measured land-use diversity using the Gini-Simpson Diversity Index [41]. The Gini-Simpson Diversity Index is given by 1—λ, where λ equals the probability that two entities taken at random from the dataset of interest represent the same type. In situations with only one class (complete homogeneity) the Gini-Simpson index would have a value equal to zero. Such diversity indices have been used to measure land-use diversity [42]. We used the following five land-use classes: annual crops, perennial crops, forests, aquaculture and built-up land (including miscellaneous uses) for which data were collected in the 2006 Agricultural Census. The area under the last class was calculated as the difference between the total area and the sum of the first four classes.
- Chicken, duck and geese flock size diversities: We used Gini-Simpson’s Diversity index based on the flock size classes reported in the Agricultural Census: 1–50, 51–150, 151–2000 and > 2000. Previous studies have shown that in Viet Nam, typical backyard flock sizes are 1–50 animals and typical flock sizes in contract poultry operations are > 2000 [43]. The contract poultry owners are small commercial enterprises.

### Other Study Variables

The following variables are listed according to their role in disease introduction, transmission and persistence, though some of these factors may have multiple roles.

- Human population related transmission.
**○** Human population density [11, 14–16, 18, 19, 44, 45].
- Poultry trade and market.
**○** Towns and cities were assumed to be active trading places [10, 18, 37, 44, 46]. So, the distance to the nearest town/city was used as indicator of poultry trade.
**○** Trade is facilitated by access to transportation infrastructure [37, 47, 48]. So, the distance to the nearest a) national highway and b) provincial highway was used as indicator of transportation infrastructure.
- Disease introduction and amplification.
**○** The densities of chicken were calculated based on commune area [15, 19, 37, 49].
- Intermediate hosts.
**○** Duck and geese densities were calculated using total commune area [11, 19, 49].
**○** As previous studies have shown a link between scavenging in rice fields by ducks and outbreaks, we also calculated duck density using only the area under rice.
- Agro-ecological and environmental risk factors.
**○** Previous studies have shown that the extent of rice cultivation is a risk factor, mainly due its association with free ranging ducks acting as scavengers [10]. We used percentage of land under rice cultivation as a measure of extent.
**○** Rice cropping intensity is also a known risk factor [11, 17, 37]. We used the mean number of rice crops per year as a measure of intensity.
  The extent of aquaculture is a known risk factor [10], possibly because water bodies offer routes for transmission and persistence of the virus. The percentage of land under aquaculture was used as a metric.
**○** Proximity to water bodies increases the risk of outbreaks [47, 50–52], possibly by increasing the chance of contact between wild water birds and domestic poultry. We measured the distance between the commune and the nearest: a) lake and b) river.
**○** Climatic variables—annual mean temperature and annual precipitation—have been associated with significant changes in risk [48, 53].
  Elevation, which is associated with types of land cover and agriculture, has been shown to be a significant risk factor in Vietnam [10].
**○** Compound Topographical Index (CTI, also known as Topographical Wetness Index) is a measure of the tendency for water to pool. Studies in Thailand and elsewhere [54] have shown that the extent of surface water is a strong risk factor, possibly due to the role of water in long-range transmission and persistence of the virus. In the absence of reliable and inexpensive data on the extent of surface water we used CTI as a proxy. CTI has been used in Ecological Niche Models (ENM) of HPAI H5N1 [55, 56]. However, given the nature of ENM studies, the effect of CTI as a risk factor has been unknown so far. CTI has been used as a risk factor in the study of other infectious and non-infectious diseases [57]. Some studies have shown that at local scales, the slope of the terrain (a component of CTI) was significantly correlated with reservoir species dominance [58]. CTI is a function of both the slope and the upstream contributing area per unit width orthogonal to the flow direction. CTI is computed as follows: CTI = ln (A_s_ / (tan (β)) where; A_s_ = Area Value calculated as ((flow accumulation + 1) * (pixel area in m^2^)) and β is the slope expressed in radians [59].
**○** Though previous studies have indicated that Normalized Difference Vegetation Index (NDVI) is a risk factor [10, 20, 55, 60, 61], we did not include it explicitly in our models, as the urban classification index we used included NDVI [9].

### Data sources

We obtained commune level data on HPAI H5N1 outbreaks from the publicly available database of the Department of Animal Health [10]. Viet Nam experienced its first major epidemic waves between December 2003 and February 2006 [10]. We chose to study the first wave (Wave 1) that ended in February 2004 and the second wave (Wave 2) that occurred between December 2004 and April 2005. In Wave 1, 21% of the communes and in Wave 2, 6% of the communes experienced outbreaks.

We used data from the 1999 Population Census of Viet Nam to estimate human population per commune. We relied on data from two Agriculture Censuses of Viet Nam. This survey is conducted every five years covering all rural households and those peri-urban households that own farms. Thus about three-fourths of all of the country’s households are included. The contents of the survey include number of households in major production activities, population, labor classified by sex, age, qualification, employment and major income source; agriculture, forestry and aquaculture land used by households classified by source, type, cultivation area for by crop type; and farming equipment by purpose. Commune level surveys include information on rural infrastructure, namely electricity, transportation, medical stations, schools; fresh water source, communication, markets, etc. Detailed economic data are collected for large farms. We used the 2006 Agriculture Census for most variables because the first three epidemic waves occurred between the Agricultural Censuses of 2001 and 2006 but were closer in time to the 2006 census [10]. However, for data on poultry numbers we used the 2001 Agriculture Census data set because between 1991 and 2003 the poultry population grew at an average rate of 7% annually. However, in 2004, after the first wave of the H5N1 epidemic, the poultry population fell 15%. Only by mid-2008 did the poultry population return close to pre-epidemic levels. Thus, we considered the poultry population data from the 2001 census to be more representative. We aggregated census household data to the commune level. A three-way classification of the rural-to-urban transition was based on a related study [9].

Raster data on annual mean temperature and precipitation were obtained from the WorldClim database and converted to commune level data. The bioclimatic variables were compiled from the monthly temperature and precipitation values and interpolated to surfaces at 90m spatial resolution [62]. This public database provides data on the average climatic conditions of the period 1950–2000.

Elevation was generated from SRTM 90 meter Digital Elevation Models (DEM) acquired from the Consortium for Spatial Information (*CGIAR*-*CSI*). Compound Topographical Index (CTI) data were generated using the Geomorphometry and Gradient Metrics Toolbox for ArcGIS 10.1.

### Data pre-processing and collinearity

Prior to risk factor analysis we cleaned the data by identifying illogical values for all variables and then either assigning a missing value to them or adjusting the values. Illogical values occurred mainly (less than 1% of the cases) for land-related variables such as percentage of commune land under a particular type of land use. Next we tested each variable for normality using the BestFit software (Palisade Corporation). Most of the variables were found to follow a log-normal distribution and a log-transform was used on them. We then examined the bi-variate correlations between all the risk factors (or their log-transform, as the case may be). Correlations were analyzed separately for each place. Certain risk factors were then eliminated from consideration when |r| ≥ 0.5 (r is the Pearson correlation coefficient). When two risk factors were highly correlated, we chose to include the one which had not been adequately studied explicitly in previously published risk models. Notably, we excluded a) elevation (correlated with human population density, chicken density, duck density, percentage land under paddy, annual temperature and compound topographical index), b) human population density (correlated with elevation and CTI), c) chicken density (only at national level, correlated with CTI), d) duck and goose density (correlated with elevation, chicken density, percentage land under paddy, land use diversity index and CTI), e) annual temperature (correlated with elevation and CTI) and f) cropping intensity (correlated with percentage land under paddy).

### Analysis

Considering the importance of spatial autocorrelation in such epidemics, we used two modeling approaches: 1) multi-level Generalized Linear Mixed Model (GLMM) and 2) Boosted Regression trees (BRT) [63, 64] with an autoregressive term [65]. GLMM is a ‘place’ oriented approach that is well suited to analyzing the effect of administrative groupings, while BRT is a ‘space’ oriented approach that accounts for the effects of physical proximity. We began by deriving an autoregressive term by averaging the presence/absence among a set of neighbors defined by the limit of autocorrelation, weighted by the inverse of the Euclidean distance [65]. The limit of the autocorrelation of the response variable was obtained from the range of the spatial correlogram ρ (h) [66]. To determine which predictor variables to include in the two models, we conducted logistic regression modeling separately for each of them one by one but included the autoregressive term each time. We finally included only those variables whose coefficient had a significance value p ≤0.2 (in at least one wave-place combination) and we noted the sign of the coefficient. This choice of p value for screening risk factors is common in similar studies [15, 18, 45, 67].

We used a two-level GLMM (communes nested under districts) to take account of random effects for an area influenced by its neighbors, and thus, we studied the effect of spatial autocorrelation. We used robust standard errors for tests of fixed effects. Boosted regression trees, also known as stochastic gradient boosting, was performed to predict the probability of HPAI H5N1 occurrence and determine the relative influence of each risk factor to the HPAI H5N1 occurrence. This method was developed recently and applied widely for distribution prediction in various fields of ecology [63, 64]. It is widely used for species distribution modeling where only the sites of occurrence of the species are known [68]. The method has been applied in numerous studies for predicting the distribution of HPAI H5N1 disease [16, 51, 69–71]. BRT utilizes regression trees and boosting algorithms to fit several models and combines them for improving prediction by performing iterative loop throughout the model [63, 64].

The advantage of BRT is that it applies stochastic processes that include probabilistic components to improve predictive performance. We used regression trees to select relevant predictor variables and boosting to improve accuracy in a single tree. The sequential process allows trees to be fitted iteratively through a forward stage-wise procedure in the boosting model. Two important parameters specified in the BRT model are learning rate (lr) and tree complexity (tc) to determine the number of trees for optimal prediction [63, 64]. In our model we used 10 sets of training and test points for cross-validation, a tree complexity of 5, a learning rate of 0.01, and a bag fraction of 0.5. Other advantages of BRT include its insensitivity to co-linearity and non-linear responses. However, for the sake of consistency with the GLMM method, we chose to eliminate predictors that were highly correlated with other predictors and to make log-transforms where needed. In the GLMM models we used p ≤ 0.05 to identify significant risk factors.

The predictive performances of the models were assessed by the area under the curve (AUC) of the receiver operation characteristic (ROC) curve. AUC is a measure of the overall fit of the model that varies from 0.5 (chance event) to 1.0 (perfect fit) [72]. A comparison of AUC with other accuracy metrics concluded that it is the most robust measure of model performance because it remained constant over a wide range of prevalence rates [73]. We used the corrected Akaike Information Criteria (AICc) to compare each GLMM model with and without its respective suite of fixed predictors.

We used SPSS version 21 (IBM Corp., New York, 2012) for GLMM and R version 3.1.0 (The R Foundation for Statistical Computing, 2014) for the BRT. For calculating the spatial correlogram we used the spdep package of R.

## Results

The fourteen predictor variables we modeled (see tables) were all found to be significantly associated with HPAI H5N1 outbreaks (p ≤ 0.2) in at least one wave-place combination based on univariate analysis (but including the autoregressive term) (Table 1). Land-use diversity, chicken density, poultry flock size diversity and distance to national highway were found to have significant associations across five of the six wave-place combinations.

**Table 1:** Unadjusted coefficients (β) for the final set of predictors based on autologistic regression.

Predictor	Wave 1 (December ‘03 –February ‘04)			Wave 2 (December ‘04 –April ‘05)		
	Viet Nam	Red River Delta	Mekong River Delta	Viet Nam	Red River Delta	Mekong River Delta
Urbanicity: rural^†^	00.000^#^	00.027	00.462	00.000	00.027	00.683
Urbanicity: peri-urban	0.3220.000	0.2850.011	-0.1050.507	0.5910.000	0.6560.007	0.0270.871
Urbanicity: urban	0.2310.112	-0.1860.571	0.3780.321	-0.0770.792	0.0850.909	-0.3530.401
Percentage land under rice*	2.1250.000	1.4540.084	0.2150.770	5.6330.000	1.9370.346	5.6460.000
Percentage land under aquaculture*	1.5350.086	0.9120.739	4.6300.000	-1.1150.503	4.2800.438	-4.3140.019
Land-use diversity (Gini-Simpson index)	0.8020.000	1.3990.000	0.6780.107	1.2160.000	0.7590.383	1.3450.007
Chicken density*	0.3990.000	0.4950.000	0.0300.747	0.5360.000	1.1580.015	0.4890.000
Duck-rice area density	0.0600.511	-0.2880.743	0.2470.223	0.1050.059	-14.2220.558	-0.8800.341
Chicken flock size diversity (Gini-Simpson Index)	2.2300.000	3.8430.000	-0.2110.770	1.2950.032	3.5230.012	1.7410.046
Duck & goose flock size diversity (Gini-Simpson Index)	0.6310.004	0.9590.068	0.0000.994	2.2750.000	2.3930.005	2.8460.000
Annual precipitation*	1.2870.001	6.6990.172	3.0800.015	0.1610.823	13.7430.184	0.2340.834
Compound Topographical Index*	3.8900.000	-1.5610.660	-3.9120.619	6.3660.000	16.9590.116	-6.0190.504
Shortest distance to nearest national highway*	-0.0200.318	-0.0410.161	0.0630.061	-0.0390.169	-0.1840.006	-0.0400.260
Shortest distance to nearest provincial highway*	-0.0410.009	-0.0200.436	0.0000.919	-0.1190.000	-0.1140.140	-0.0650.074
Shortest distance to nearest town*	-0.0090.683	0.1570.002	0.0600.181	-0.0730.044	0.0550.607	-0.0730.120
Shortest distance to nearest lake*	-0.0740.009	0.0580.433	-0.0100.591	0.0690.330	-0.1410.298	0.0610.629

^†^ Reference level,

* Transform of the type log_10_(1+x) was used,

^#^p values

Both the final multivariate GLMM and BRT model results for the previously studied variables are in agreement with the associations reported by others (Tables 2–7). The predictive power of the GLMM models, as measured by the AUC, is very good with AUC values ranging from 0.802 to 0.952 (Tables 2–7). The predictive power of the national models was higher than that of the delta models. The predictive power of the BRT models is good, with AUCs ranging from 0.737 to 0.914. The BRT models also had a better predictive power at the national level than at the delta level. These values are higher than those reported for Wave 1 (AUC = 0.69) and Wave 2 (AUC = 0.77) by Gilbert et al. [11]. Both Gilbert et al. [11] and this study found that at the national level the predictive performance for Wave 2 was higher than that for Wave 1. Wave 2 mainly affected the Mekong River Delta. Previous studies indicated the duck density was an important predictor [11]; our results, however, indicated that the diversity of duck flock size was a more important predictor than duck density.

**Table 2:** Model results for Viet Nam, Wave 1 (December ‘03 –February ‘04).

	GLMM			BRT		
	Coefficient	p	s.e.	Relative Influence (%)	s.e.	Rank
Intercept	-6.633	0.001	4.21	n/a		
Urbanicity				0.63	0.03	11
Urbanicity: rural	0					
Urbanicity: peri-urban	0.061	0.627	0.13			
Urbanicity: urban	0.419	0.108	0.26			
Land-use diversity (Gini-Simpson index)	0.779	0.032	0.36	1.38	0.04	5
Duck-rice area density	0.086	0.006	0.03	1.19	0.03	6
Chicken flock size diversity (Gini-Simpson Index)	1.706	0.003	0.57	5.58	0.03	1
Duck & goose flock size diversity (Gini-Simpson Index)	0.439	0.164	0.32	1.72	0.09	3
Percentage land under rice*	2.193	0.021	0.95	1.17	0.10	7
Percentage land under aquaculture*	2.143	0.245	1.84	1.43	0.09	4
Annual precipitation*	-1.967	0.083	1.14	2.35	0.10	2
Compound Topographical Index*	9.874	0.000	2.12	0.78	0.09	8
Shortest distance to nearest national highway*	0.006	0.809	0.03	0.13	0.09	13
Shortest distance to nearest provincial highway*	-0.015	0.499	0.02	0.37	0.04	12
Shortest distance to nearest town*	-0.057	0.183	0.04	0.72	0.09	9
Shortest distance to nearest lake*	-0.052	0.270	0.05	0.64	0.10	10
Autoregressive term	n/a			81.91	0.04	
AUC-ROC	0.907			Trg = 0.856, Eval = 0.839		

* Transform of the type log_10_(1+x) was used

s.e. = standard error, Rank = rank of relative influence excluding the rank of the autoregressive term, AUC-ROC = Area Under the Curve of the Receiver Operating Characteristic, Trg = Training, Eval = Evaluation

**Table 3:** Model results for Viet Nam, Wave 2 (December ‘04 –April ‘05).

	GLMM			BRT		
	Coefficient	p	s.e	Relative Influence (%)	s.e.	Rank
Intercept	-6.700	0.355	7.24			
Urbanicity				0.70	0.07	13
Urbanicity: rural	0					
Urbanicity: peri-urban	0.219	0.212	0.18			
Urbanicity: urban	0.179	0.757	0.58			
Land-use diversity (Gini-Simpson index)	1.885	0.002	0.61	1.44	0.11	8
Duck-rice area density	0.205	0.065	0.11	4.11	0.11	4
Chicken flock size diversity (Gini-Simpson Index)	1.023	0.189	0.78	4.98	0.07	3
Duck & goose flock size diversity (Gini-Simpson Index)	2.245	0.000	0.49	5.36	0.05	1
Percentage land under rice*	5.322	0.000	1.31	5.32	0.10	2
Percentage land under aquaculture*	1.852	0.536	3.00	2.73	0.10	5
Annual precipitation*	-4.716	0.001	1.41	2.50	0.05	6
Compound Topographical Index*	11.487	0.002	3.68	1.68	0.07	7
Shortest distance to nearest national highway*	-0.031	0.389	0.04	1.80	0.10	9
Shortest distance to nearest provincial highway*	-0.114	0.001	0.04	1.20	0.10	11
Shortest distance to nearest town*	-0.128	0.052	0.07	1.31	0.10	10
Shortest distance to nearest lake*	1.058	0.014	0.43	0.77	0.05	12
Autoregressive term	n/a			66.09	0.10	
AUC-ROC	0.952			Trg = 0.935, Eval = 0.913		

* Transform of the type log_10_(1+x) was used

s.e. = standard error, Rank = rank of relative influence excluding the rank of the autoregressive term, AUC-ROC = Area Under the Curve of the Receiver Operating Characteristic, Trg = Training, Eval = Evaluation

**Table 4:** Model results for Red River Delta, Wave 1 (December ‘03 –February ‘04).

	GLMM			BRT		
	Coefficient	p	s.e.	Relative Influence (%)	s.e.	Rank
Intercept	108.702	0.001	33.225	n/a		
Urbanicity				0.91	0.04	13
Urbanicity: rural	0					
Urbanicity: peri-urban	0.009	0.986	0.518			
Urbanicity: urban	0.179	0.383	0.205			
Land-use diversity (Gini-Simpson index)	0.959	0.338	1.000	4.26	0.04	8
Chicken density*	0.970	0.012	0.385	8.28	0.06	2
Duck-rice area density	-6.879	0.129	4.525	6.07	0.04	5
Chicken flock size diversity (Gini-Simpson Index)	2.424	0.073	1.352	10.62	0.05	1
Duck & goose flock size diversity (Gini-Simpson Index)	0.051	0.920	0.511	5.61	0.05	6
Percentage land under rice*	-1.643	0.362	1.802	5.03	0.06	7
Percentage land under aquaculture*	-0.974	0.799	3.832	6.30	0.04	3
Annual precipitation*	-32.626	0.003	11.114	6.21	0.09	4
Compound Topographical Index*	-7.193	0.355	0.119	3.36	0.04	10
Shortest distance to nearest national highway*	-0.079	0.082	0.045	1.59	0.06	12
Shortest distance to nearest provincial highway*	-0.027	0.472	0.038	0.49	0.09	14
Shortest distance to nearest town*	0.173	0.034	0.082	2.27	0.05	11
Shortest distance to nearest lake*	-0.041	0.730	0.119	3.57	0.09	9
Autoregressive term	n/a			35.43	0.04	
AUC-ROC	0.802			Trg = 0.827, Eval = 0.737		

* Transform of the type log_10_(1+x) was used

s.e. = standard error, Rank = rank of relative influence excluding the rank of the autoregressive term, AUC-ROC = Area Under the Curve of the Receiver Operating Characteristic, Trg = Training, Eval = Evaluation

**Table 5:** Model results for Red River Delta, Wave 2 (December ‘04 –April ‘05).

	GLMM			BRT		
	Coefficient	p	s.e.	Relative Influence (%)	s.e	Rank
Intercept	68.171	0.142	46.439			
Urbanicity: rural	0			2.13	0.04	13
Urbanicity: peri-urban	0.240	0.530	0.382			
Urbanicity: urban	0.041	0.033	1.245			
Percentage land under rice*	-7.892	0.046	3.944	6.81	0.09	6
Percentage land under aquaculture*	-2.552	0.688	6.362	6.31	0.07	7
Land-use diversity (Gini-Simpson index)	1.592	0.451	2.113	5.62	0.08	9
Chicken density*	1.017	0.033	0.476	10.34	0.07	2
Duck-rice area density	-70.094	0.030	32.181	7.95	0.08	4
Chicken flock size diversity (Gini-Simpson Index)	0.935	0.571	1.651	12.86	0.09	1
Duck & goose flock size diversity (Gini-Simpson Index)	1.934	0.087	1.130	8.07	0.04	3
Annual precipitation*	-32.390	0.038	15.625	7.85	0.28	5
Compound Topographical Index*	26.477	0.258	23.375	4.66	0.28	10
Shortest distance to nearest national highway*	-0.081	0.294	0.077	2.56	0.07	12
Shortest distance to nearest provincial highway*	-0.136	0.085	0.079	0.96	0.08	14
Shortest distance to nearest town*	0.093	0.619	0.188	2.72	0.09	11
Shortest distance to nearest lake*	-0.262	0.054	0.136	6.27	0.04	8
Autoregressive term	n/a			14.9	0.28	
AUC-ROC	0.902			Trg = 0.987, Eval = 0.755		

* Transform of the type log_10_(1+x) was used

s.e. = standard error, Rank = rank of relative influence excluding the rank of the autoregressive term, AUC-ROC = Area Under the Curve of the Receiver Operating Characteristic, Trg = Training, Eval = Evaluation

**Table 6:** Model results for Mekong River Delta, Wave 1 (December ‘03 –February ‘04).

	GLMM			BRT		
	Coefficient	p	s.e.	Relative Influence (%)	s.e.	Rank
Intercept	35.702	0.129	23.528	n/a		
Urbanicity.				1.43	0.08	14
Urbanicity: rural	0					
Urbanicity: peri-urban	0.149	0.592	0.277			
Urbanicity: urban	1.292	0.025	0.575			
Percentage land under rice*	3.632	0.021	1.571	4.32	0.06	8
Percentage land under aquaculture*	5.393	0.063	2.898	6.47	0.06	3
Land-use diversity (Gini-Simpson index)	-0.059	0.956	1.069	5.39	0.04	5
Chicken density*	-0.082	0.791	0.308	4.59	0.07	6
Duck-rice area density	0.297	0.454	0.396	4.46	0.07	7
Chicken flock size diversity (Gini-Simpson Index)	0.689	0.642	1.481	6.50	0.07	2
Duck & goose flock size diversity (Gini-Simpson Index)	-0.057	0.935	0.704	6.25	0.08	4
Annual precipitation*	-12.623	0.000	3.288	15.67	0.07	1
Compound Topographical Index*	3.183	0.863	18.482	3.52	0.08	9
Shortest distance to nearest national highway*	0.141	0.036	0.067	1.82	0.06	13
Shortest distance to nearest provincial highway*	0.012	0.825	0.054	2.06	0.07	11
Shortest distance to nearest town*	0.074	0.504	0.111	2.04	0.07	12
Shortest distance to nearest lake*	0.077	0.135	0.569	2.88	0.04	10
Autoregressive term	n/a			32.63	0.04	
AUC-ROC	0.891			Trg = 0.911, Eval = 0.811		

* Transform of the type log_10_(1+x) was used

s.e. = standard error, Rank = rank of relative influence excluding the rank of the autoregressive term, AUC-ROC = Area Under the Curve of the Receiver Operating Characteristic, Trg = Training, Eval = Evaluation

**Table 7:** Model results for Mekong River Delta, Wave 2 (December ‘04 –April ‘05).

	GLMM			BRT		
	Coefficient	p	s.e.	Relative Influence (%)	s.e.	Rank
Intercept	-10.082	0.554	17.043			
Urbanicity				0.72	0.07	14
Urbanicity: rural	0					
Urbanicity: peri-urban	0.105	0.641	0.225			
Urbanicity: urban	0.176	0.758	0.569			
Percentage land under rice*	5.009	0.001	1.529	9.04	0.05	3
Percentage land under aquaculture*	0.936	0.781	3.360	6.75	0.07	6
Land-use diversity (Gini-Simpson index)	1.956	0.018	0.823	6.85	0.07	5
Chicken density*	0.364	0.145	0.250	7.20	0.07	4
Duck-rice area density	0.597	0.239	0.507	9.70	0.08	2
Chicken flock size diversity (Gini-Simpson Index)	1.674	0.147	1.153	5.91	0.08	7
Duck & goose flock size diversity (Gini-Simpson Index)	2.533	0.000	0.704	10.21	0.07	1
Annual precipitation*	-2.719	0.277	2.498	5.80	0.05	8
Compound Topographical Index*	8.874	0.529	14.076	4.13	0.07	9
Shortest distance to nearest national highway*	-0.019	0.716	0.052	3.84	0.07	10
Shortest distance to nearest provincial highway*	-0.149	0.005	0.053	2.14	0.05	13
Shortest distance to nearest town*	-0.074	0.433	0.094	3.36	0.07	11
Shortest distance to nearest lake*	1.006	0.005	0.382	3.20	0.08	12
Autoregressive term	n/a			21.14	0.07	
AUC-ROC	0.849			Trg = 0.926, Eval = 0.763		

* Transform of the type log_10_(1+x) was used

s.e. = standard error, Rank = rank of relative influence excluding the rank of the autoregressive term, AUC-ROC = Area Under the Curve of the Receiver Operating Characteristic, Trg = Training, Eval = Evaluation

Both the GLMM and BRT models found annual precipitation to be a significant factor. The GLMM model indicated a negative association; similar to what was found by studies in China [51] and in the Red River Delta [53]. A global study of human cases also found occurrence to be higher under drier conditions [74]. Generally, the role of precipitation was found to be far more significant in the deltas than for the country as a whole.

**Fig 1:**
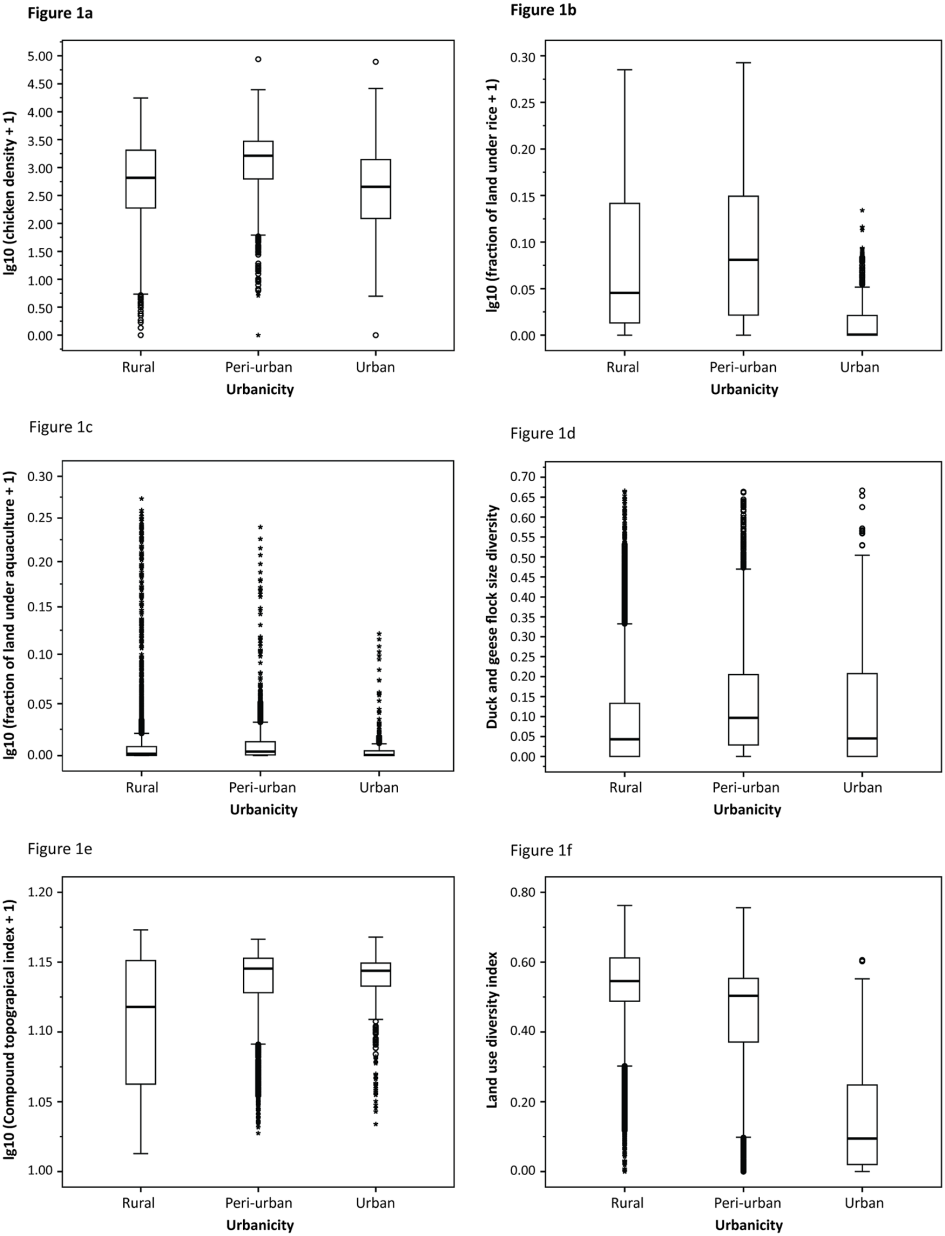
Variations in variables across urbanicity classes for all of Vietnam. a: Variation of chicken density across urbanicity. b: Variation of fraction of land under rice across urbanicity. c: Variation of fraction of land under aquaculture across urbanicity. d: Variation of duck and geese flock size diversity across urbanicity. e: Variation of CTI across urbanicity. f: Variation of land-use diversity across urbanicity.

The unadjusted Relative Risk (RR) of peri-urban areas in comparison with non-peri-urban areas was 1.41 and 1.60 for Waves 1 and 2, respectively. In terms of urbanicity, we found that chicken density, percentage of land under rice, percentage of land under aquaculture, flock size diversity for duck and geese, and the Compound Topographical Index (CTI) to be highest in peri-urban areas (Fig 1a–1e). We also found that land-use diversity was higher in rural areas, but peri-urban areas had diversity levels only marginally lower (Fig 1f). The urbanicity variable alone, however, was not found to be significantly associated with HPAI H5N1 in any place according to the GLMM model except for the urban level in Red River Delta for Wave 2 and in the Mekong River Delta for Wave 1. The BRT model ranked urbanicity as one of the least influential variables.

Land-use diversity was found to be significantly associated with HPAI H5N1 in both waves for Viet Nam according to the GLMM model, but at the delta level the association was significant only for Wave 2 in the Mekong River Delta. The BRT model indicated that land-use diversity highly influenced HPAI H5N1 at the national level in Wave 2. For the remaining wave-place combinations land-use diversity had middle to below-middle rank of influence.

Both the GLMM and BRT models indicated that the diversity of chicken flock-size had a strong association with HPAI H5N1 for both waves at the national level. This was generally found to be true at the delta levels with some exceptions. The diversity of duck and goose flock size was also significantly associated with HPAI H5N1 in all places, but the associations were much stronger in Wave 2 than in Wave 1.

The GLMM model indicated that the CTI had a very strong association with HPAI H5N1 at the national level in both waves although this was not true in the two deltas. The CTI is a steady state wetness index commonly used to quantify topographic control on hydrological processes. Accumulation numbers in flat areas, like deltas, are very large; hence the CTI was not a relevant variable in the GLMM model in these areas. The BRT model however indicated that CTI had middle to low influence in all waves and places.

We found very high spatial clustering effects as indicated by the fact that in all waves and places the BRT model found the spatial autocorrelation term to have the highest rank of influence. As expected, the relative influence of the autocorrelation term at the national level was higher (60–78%) than at the delta levels (14–35%). In the GLMM models we found the Akaike Information Criterion (AIC) using the entire set of 14 variables to be much lower than the AICs of a GLMM model without fixed effects. This indicated that though clustering effects were significant, our theory driven predictor variables improved model performance.

A limitation of using surveillance methods for the dependent variable (poultry outbreaks) is that the data may have reporting/detection biases [11]. Under-reporting/detection in rural areas as compared to peri-urban areas is possible. We believe that the urbanicity and the shortest distance to nearest town risk factors serve as rough proxies for reporting/detection efficiency. Previous studies have tended to use human population density as a proxy for this purpose. In our study we found a strong association between human population density and urbanicity. But we acknowledge that a categorical variable such as urbanicity may provide less sensitivity than a continuous variable such as human population density in this specific context.

## Discussion

This study explored the validity of a general model for disease emergence that combined the IOM ‘convergence model’ [6] and the social-ecological systems model [7, 8], for investigating the specific case of HPAI in Vietnam. We sought to test the hypotheses that measures of urbanization, land-use diversification, and poultry intensification are correlated with outbreaks in poultry. Our results generally support the hypothesis that social-ecological system transformations are associated with H5NI outbreaks in poultry.

The results presented here highlight three main findings: 1) when relevant risk factors are taken into account, urbanization is generally not a significant independent risk factor; but in peri-urban landscapes emergence factors converge, including higher levels of chicken densities, duck and geese flock size diversities, and fraction of land under rice or aquaculture; 2) high land-use diversity landscapes, a variable not previously considered in spatial studies of HPAI H5N1, are at significantly greater risk for HPAI H5N1 outbreaks; as are 3) landscapes where intensive and extensive forms of poultry production are co-located.

Only one other study has explicitly examined urbanicity in the context of HPAI H5N1. Loth et al. [17] found peri-urban areas in Indonesia were significantly associated with HPAI H5N1 cases, even based on multivariate models. Our study, however, attempted both to associate HPAI H5N1 with degree of urbanicity and to determine the features of peri-urban areas that place them at risk. When those features (i.e., chicken densities, duck and geese flock size diversities, and the fraction of land under rice or aquaculture) are included in multivariate models, the role of the urbanization variable per se diminishes. We found in the main river deltas in Viet Nam (Red River and Mekong), urbanization had no significant association with HPAI H5N1. This may be due to the fact that the deltas are more homogenous, in terms of urbanization, than the country as a whole.

This is the first study to examine land-use diversity as a risk factor for HPAI H5N1. Measured by the Gini-Simpson Diversity Index of the five land-use classes on which data were collected in the 2006 Viet Nam Agricultural Census, and the presence or absence of HPAI outbreaks at the commune level, our results indicate a strong association between land-use diversity and HPAI H5N1 at the national level and in the Mekong River Delta. This metric captures both the variety of habitats and of the complexity of geospatial patterning likely associated with transmission intensity. Our results are similar to what has been observed by studies of other EIDs using fragmentation metrics (e.g. [75–77]. This is one of the few studies, however, to link landscape fragmentation to an EID disease in poultry and not just to the vector and/or hosts of the EID.

Previous studies have focused on poultry production factors such as type of species, size of flocks, and extent of commercialization (e.g. [15, 17–19]. This study expands on those findings by providing evidence that when intensive and extensive systems of chicken and/or duck and geese production co-exist in the same commune, the commune experiences higher risk of disease outbreak. Future studies need to examine the biological causal mechanisms in this context.

We suggest that national census data (particularly agricultural censuses) compiled at local levels of administration provide valuable information that are not available from remotely sensed data (such as poultry densities) or require a large amount of labor to map at national to larger scales (land-use diversity). Mapping land-use classes at the national scale for local administrative units (i.e., the 10,820 communes in Viet Nam) is not an insignificant task. Future studies, however, could examine the correlation between a census-based metric with metrics derived from remote sensing used to measure proportional abundance of each land-cover type within a landscape [78]. Vietnam is relatively advanced in making digital national population and agricultural census data available in a format that can be linked to administrative boundaries. While other nations are beginning to develop similar capacities, in the short term the application of this method to other countries may be limited. Ultimately, both census and remotely sensed data can be used independently to map the urban transition and diversity of land use; these tools, however, may provide their greatest insights when used together.

Another important contribution of this study was the discovery of the importance of CTI. So far CTI had been used only in ecological niche modeling studies of HPAI H5N1; the specific role and direction of influence of CTI had has so far been unknown. Our study, the first to use CTI as a risk factor, found it had a large positive influence on HPAI H5N1 risk at the national level. Previous studies have highlighted the role of surface water extent in the persistence and transmission of the HPAI H5N1 virus. These studies measured surface water extent as area covered by water, magnitude of seasonal flooding, distance to the nearest body of water, or other variables that are often difficult to map using remotely sensed data, especially for large area studies. CTI on the other hand has the potential to serve as an excellent surrogate which can easily be measured in a GIS database.

The national and regional (delta) models differed quite considerably, both in terms of performance and significant risk factors. In the deltas we commonly found only chicken density, duck flock size diversity and annual precipitation to be significant. This suggests dynamics of risk at the commune level are strongly dependent on the spatial range of analysis, consistent with another study in the Mekong Delta [61]. Though that study’s model initially included three dozen commonly known risk factors, the significant risk factors were limited to poultry flock density, proportion households with electricity, re-scaled NDVI median May-October, buffalo density and sweet potato yield. Another study in the Red River Delta [79] found that in addition to the typical poultry density metrics, only the presence of poultry traders was significant. We speculate that for smaller regions, especially for known hot-spots, the relevant risk factors are those that reflect short-range, short-term driving forces such as poultry trading, presence of live bird markets and wet markets etc. Improving model performance for smaller regions would require highly refined and nuanced metrics for poultry trading, road infrastructure, water bodies, etc.—data that are typically not available through census surveys. The differences between the national and regional models suggest that our results can inform planners making decisions at different hierarchical levels of jurisdiction: national, region and local.

Our study has the potential to inform the design of future research related to the epidemiology of other EIDs in Viet Nam and elsewhere. For example, we speculate that in Southeast Asia, Japanese encephalitis, the transmission of which is associated with rice cultivation and flood irrigation [80], may also show a strong association with peri-urbanization. In some areas of Asia these ecological conditions occur near, or occasionally within, urban centers. Likewise, Hantaan virus, the cause of Korean hemorrhagic fever, is associated with the field mouse *Apodemus agrarius* and rice harvesting in fields where the rodents are present [80]. Our work has demonstrated that the percentage of land under rice in peri-urban areas and rural areas is similar. Hence diseases associated with rice production are likely to peak in peri-urban areas given other risk factors such as land-use diversity, CTI, and distance to infrastructure. Our poultry flock-size diversity findings may also be relevant to understanding the dynamics of other poultry related infections such as Newcastle disease.

Finally, these results suggest the validity of a general model of zoonotic disease emergence that integrates IOM’s convergence model with the subsequently proposed social-ecological systems and EID framework. Thus, convergence represents the coalescence in time and space of processes associated with land-cover and land-use changes. Project results question whether the urban/rural land-use dichotomy is useful when large areas and parts of the population are caught between the two. Planners need better tools for mapping the rural-urban transition, and for understanding how the specific nature of peri-urban environments creates elevated health risk that require adaptation of existing planning, land use, and development practices.
