# Correction: Evidence for the Convergence Model: The Emergence of Highly Pathogenic Avian Influenza (H5N1) in Viet Nam

**DOI:** 10.1371/journal.pone.0141519

**Published:** 2015-10-20

**Authors:** The PLOS ONE Staff

There is an error in Table 1. The publisher apologizes for the error. Please see the corrected Table 1 here.

**Table 1:** Unadjusted coefficients (β) for the final set of predictors based on autologistic regression.

Predictor	Wave 1			Wave 2		
	(December ‘03–February ‘04)			(December ‘04–April ‘05)		
	Viet Nam	Red River Delta	Mekong River Delta	Viet Nam	Red River Delta	Mekong River Delta
Urbanicity: rural^†^	0	0	0	0	0	0
	0.000^#^	0.027	0.462	0.000	0.027	0.683
Urbanicity: peri-urban	0.322	0.285	-0.105	0.591	0.656	0.027
	0.000	0.011	0.507	0.000	0.007	0.871
Urbanicity: urban	0.231	-0.186	0.378	-0.077	0.085	-0.353
	0.112	0.571	0.321	0.792	0.909	0.401
Percentage land under rice*	2.125	1.454	0.215	5.633	1.937	5.646
	0.000	0.084	0.770	0.000	0.346	0.000
Percentage land under aquaculture*	1.535	0.912	4.630	-1.115	4.280	-4.314
	0.086	0.739	0.000	0.503	0.438	0.019
Land-use diversity (Gini-Simpson index)	0.802	1.399	0.678	1.216	0.759	1.345
	0.000	0.000	0.107	0.000	0.383	0.007
Chicken density*	0.399	0.495	0.030	0.536	1.158	0.489
	0.000	0.000	0.747	0.000	0.015	0.000
Duck-rice area density	0.060	-0.288	0.247	0.105	-14.222	-0.880
	0.511	0.743	0.223	0.059	0.558	0.341
Chicken flock size diversity (Gini-Simpson Index)	2.230	3.843	-0.211	1.295	3.523	1.741
	0.000	0.000	0.770	0.032	0.012	0.046
Duck & goose flock size diversity (Gini-Simpson Index)	0.631	0.959	0.000	2.275	2.393	2.846
	0.004	0.068	0.994	0.000	0.005	0.000
Annual precipitation*	1.287	6.699	3.080	0.161	13.743	0.234
	0.001	0.172	0.015	0.823	0.184	0.834
Compound Topographical Index*	3.890	-1.561	-3.912	6.366	16.959	-6.019
	0.000	0.660	0.619	0.000	0.116	0.504
Shortest distance to nearest national highway*	-0.020	-0.041	0.063	-0.039	-0.184	-0.040
	0.318	0.161	0.061	0.169	0.006	0.260
Shortest distance to nearest provincial highway*	-0.041	-0.020	0.000	-0.119	-0.114	-0.065
	0.009	0.436	0.919	0.000	0.140	0.074
Shortest distance to nearest town*	-0.009	0.157	0.060	-0.073	0.055	-0.073
	0.683	0.002	0.181	0.044	0.607	0.120
Shortest distance to nearest lake*	-0.074	0.058	-0.010	0.069	-0.141	0.061
	0.009	0.433	0.591	0.330	0.298	0.629

^†^ Reference level,

* Transform of the type log_10_(1+x) was used,

^#^p values
